# Zika Virus infection of rhesus macaques leads to viral persistence in multiple tissues

**DOI:** 10.1371/journal.ppat.1006219

**Published:** 2017-03-09

**Authors:** Alec J. Hirsch, Jessica L. Smith, Nicole N. Haese, Rebecca M. Broeckel, Christopher J. Parkins, Craig Kreklywich, Victor R. DeFilippis, Michael Denton, Patricia P. Smith, William B. Messer, Lois M. A. Colgin, Rebecca M. Ducore, Peta L. Grigsby, Jon D. Hennebold, Tonya Swanson, Alfred W. Legasse, Michael K. Axthelm, Rhonda MacAllister, Clayton A. Wiley, Jay A. Nelson, Daniel N. Streblow

**Affiliations:** 1 Vaccine and Gene Therapy Institute, Oregon Health and Science University, Beaverton, Oregon, United States of America; 2 Department of Molecular Microbiology and Immunology, Oregon Health and Science University, Portland, Oregon, United States of America; 3 Pathology Services Unit, Division of Comparative Medicine, Oregon National Primate Research Center, Beaverton, Oregon, United States of America; 4 Division of Reproductive & Developmental Sciences, Oregon National Primate Research Center, Beaverton, Oregon, United States of America; 5 Department of Obstetrics & Gynecology, Oregon Health and Science University, Portland, Oregon, United States of America; 6 Division of Pathobiology and Immunology, Oregon National Primate Research Center, Beaverton, Oregon, United States of America; 7 Clinical Medicine Unit, Division of Comparative Medicine, Oregon National Primate Research Center, Beaverton, Oregon, United States of America; 8 UPMC Presbyterian Hospital, Division of Neuropathology, University of Pittsburgh, Pittsburgh, Pennsylvania, United States of America; University of Texas Medical Branch, UNITED STATES

## Abstract

Zika virus (ZIKV), an emerging flavivirus, has recently spread explosively through the Western hemisphere. In addition to symptoms including fever, rash, arthralgia, and conjunctivitis, ZIKV infection of pregnant women can cause microcephaly and other developmental abnormalities in the fetus. We report herein the results of ZIKV infection of adult rhesus macaques. Following subcutaneous infection, animals developed transient plasma viremia and viruria from 1–7 days post infection (dpi) that was accompanied by the development of a rash, fever and conjunctivitis. Animals produced a robust adaptive immune response to ZIKV, although systemic cytokine response was minimal. At 7 dpi, virus was detected in peripheral nervous tissue, multiple lymphoid tissues, joints, and the uterus of the necropsied animals. Notably, viral RNA persisted in neuronal, lymphoid and joint/muscle tissues and the male and female reproductive tissues through 28 to 35 dpi. The tropism and persistence of ZIKV in the peripheral nerves and reproductive tract may provide a mechanism of subsequent neuropathogenesis and sexual transmission.

## Author summary

Although it was first identified almost 70 years ago, Zika virus had rarely been associated with pathology in humans until the 21^st^ century. Recent outbreaks in the South Pacific and the Americas have been characterized by numerous confirmed cases, some involving neurologic sequelae and, of most concern, birth defects following infection of pregnant women. Here, we present the results of experimental infection of adult rhesus macaques with a strain of Zika virus isolated during the recent epidemic in the Western hemisphere. Following infection, Zika virus was detected in the sera and urine of all infected animals. Further, we detected virus in multiple tissues of infected animals as late as 35 days post infection, indicating viral persistence. The apparent tropism of the virus for tissues of the peripheral nervous system as well as the reproductive tracts of males and females has implications for the further characterization of the mechanism(s) of Zika virus pathogenesis. Additionally, this model provides a platform for development and testing of preventative or therapeutic interventions to combat the emergence of this virus.

## Introduction

Zika virus (ZIKV), once a little-studied member of the family *Flaviviridae*, forcefully emerged across the Western Hemisphere in 2015–16. As of October, 2016, the Centers for Disease Control and Prevention (CDC) lists 60 countries worldwide, including the continental U.S., that have reported autochthonous transmission of the virus, primarily through an *Aedes spp* mosquito vector (https://www.cdc.gov/zika/geo/active-countries.html). The virus is also believed to be endemic in multiple countries in Africa and Southeast Asia. The World Health Organization (WHO) has estimated that 3–4 million individuals will be infected with ZIKV in the next year [1]. While an estimated 80% of all infections are asymptomatic or subclinical, the remaining 20% of ZIKV infections often resembles infection by co-circulating dengue (DENV) or chikungunya (CHIKV) viruses, with typical symptoms including fever, rash, headache and arthralgia, although ZIKV does appear to have a fairly distinctive association with conjunctivitis [2,3]. ZIKV was first isolated in 1947, from serum taken from a febrile sentinel rhesus macaque (RM) in the Zika Forest region of Uganda [4,5]. Although reported instances of human ZIKV-associated disease during the 20^th^ century had been sporadic with generally mild disease, large outbreaks were reported in Yap State, Micronesia in 2007 and in French Polynesia in 2013, resulting in ~900 and 30,000 symptomatic cases, respectively [6,7]. Illness during these outbreaks was initially characterized as self-limiting and did not require hospitalization. However, 74 patients in French Polynesia who experienced confirmed or probable ZIKV infection later presented with neurological complications. Over half of these were characterized as Guillain-Barré syndrome (GBS); the remainder included various encephalitides, paraesthesia, facial paralysis and myelitis. Similarly, increases in GBS have been reported in 12 countries worldwide, together with laboratory confirmation of ZIKV infection associated with these cases [8]. Of particular concern is the presumed causal relationship between ZIKV and microcephaly in developing fetuses. In several cases, ZIKV infection of the mother during pregnancy, as well as the presence of ZIKV in the amniotic fluid or tissue of fetuses showing evidence of microcephaly was reported [9–12]. However, the specific mechanisms by which ZIKV causes fetal neurological defects in humans remain unknown. Mouse models, both with and without intact innate immune signaling, of ZIKV infection during pregnancy have proven susceptible to infection of placental and fetal tissue, resulting in intrauterine growth restriction and fetal death [13,14]. However, differences in placental architecture and fetal development in the mouse vis-à-vis humans suggest that certain aspects of ZIKV pathogenesis during pregnancy may not be reflected in the murine infection model [15].

Non-human primates, by virtue of their relatedness to humans, are valuable models for the study of human disease. For example, experimental infection of RM with yellow fever virus (YFV) results in viscerotropic disease that closely parallels the course observed in humans [16,17]. Additionally, we have recently developed a model of CHIKV infection in the RM that recapitulates several aspects of human disease, including joint tropism and inflammation, viremia, and robust innate and adaptive immune responses [18,19]. Both DENV [20] and WNV [21] infection of RMs results in detectable viremia and immune response, although infection is not associated with overt pathology. Prior to the current epidemic, the outcome of ZIKV infection in RM model had not been well characterized. However, the fact that the virus was originally isolated from a febrile RM suggests that viral replication, immune response, and aspects of pathogenesis may be modeled in RMs. Here, we report outcomes of infection of adult RMs with a ZIKV strain currently circulating in the Western hemisphere (a 2015 isolate from Puerto Rico). Sub-cutaneous inoculation of animals produced transient, detectable viremia and viruria, as well as clinical symptoms described in human ZIKV infections (e.g. fever, rash, conjunctivitis). These data are comparable to those additional recently published studies of ZIKV infection in RM [22–24]. Herein we also extend these data by examination of tissue tropism during infection. Cohorts of animals necropsied at day 7, 28 or 35 pi indicated that viral RNA was present within secondary lymphoid tissues, joints, peripheral nervous tissue and organs of the female reproductive tract. Further, a robust immune response, including production of neutralizing antibodies, was observed in all infected animals. Our data suggest that RM may provide a useful model for the study of ZIKV pathogenesis, as well as a platform for the testing of vaccines or anti-viral therapeutics.

## Materials and methods

### Ethics statement regarding non-human primate research

All Zika virus infection experiments utilizing animals were performed in compliance with guidelines established by the Animal Welfare Act for housing and care of laboratory animals and conducted in accordance with Oregon National Primate Research Center (ONPRC) Institutional Animal Care and Use Committee approved protocol (IACUC #0993). RM studies were performed in ABSL-3 or ABSL-2 containment facilities at the Oregon National Primate Research Center (ONPRC), which are accredited by the Assessment and Accreditation of Laboratory Animal Care (AAALAC) International. Appropriate procedures were utilized in order to reduce potential distress, pain and discomfort. Ketamine (10 mg/kg) was used to sedate the animals during all procedures including routine blood draws performed by trained veterinary staff. Rhesus monkeys were fed standard monkey chow twice daily and the amount was matched to each animal according to body weight, age and sex and intake as monitored. Animals also received daily food supplements and other enrichment devices. The infected animals were caged with partners or caged separately but within visual and auditory contact of other animals in order to promote social behavior. At the designated time points, the animals were euthanized according to the recommendations of the American Veterinary Medical Association 2013 panel on Euthanasia.

### Cells and viruses

Zika virus train PRVABC59 was isolated by the Centers for Disease Control (CDC) from an individual in Puerto Rico in December 2015 [25]. PRVABC59 was obtained from the CDC, and passaged twice in C6/36 cells (American Type Culture Collection, ATCC). To prepare virus stock, infected C6/36 tissue culture supernatant was concentrated through a 20% sorbitol cushion and titered in Vero cells (ATCC) using a focus-formation assay. The virus stock was sequenced and found to conform to the previously described sequence (Genbank accession #KU501215.1) with the following four single base pair substitutions: G-1964-T (Envelope protein V to L; frequency 82.7%); T-3147-C (NS2A protein M to T; frequency 14.5%); C-5676-T (NS3 protein S to F; frequency 36.8%); and C-7915-T (NS5 protein silent mutation; frequency 13.5%). All cells were cultured in Dulbecco’s Modified Eagle Medium (DMEM; Corning) containing penicillin-streptomycin-glutamine (PSG; Corning) and 5–10% fetal calf serum (FCS; HyClone). Vero cells were grown at 37°C and C6/36 cells were grown at 28°C.

### Focus forming assay

Serial dilutions of virus were plated in 96-well plates seeded with Vero cells, allowed to adsorb for 1 h, followed by overlay with 0.5% carboxymethyl-cellulose (CMC; Sigma). At 30 h pi, cells were fixed with 4% paraformaldehyde, washed twice with PBS and blocked/ permeabilized for 1 h in PBS supplemented with 2% normal goat serum (NGS; Sigma) and 0.4% triton X-100. Cells were then washed twice with PBS followed by incubation with 0.3 μg/ml anti-flavivirus monoclonal antibody 4G2 [26] in PBS supplemented with 2% NGS for 1 h, washed twice more with PBS, incubated with anti-mouse IgG-horseradish peroxidase (Santa Cruz Biotech) for 1 h, and washed twice with PBS. Foci were visualized by incubation with the Vector VIP peroxidase substrate kit (Vector Labs) according to manufacturer’s specifications and counted using an ELIspot reader (AID).

### Nonhuman primate infection

**Fig 1:**
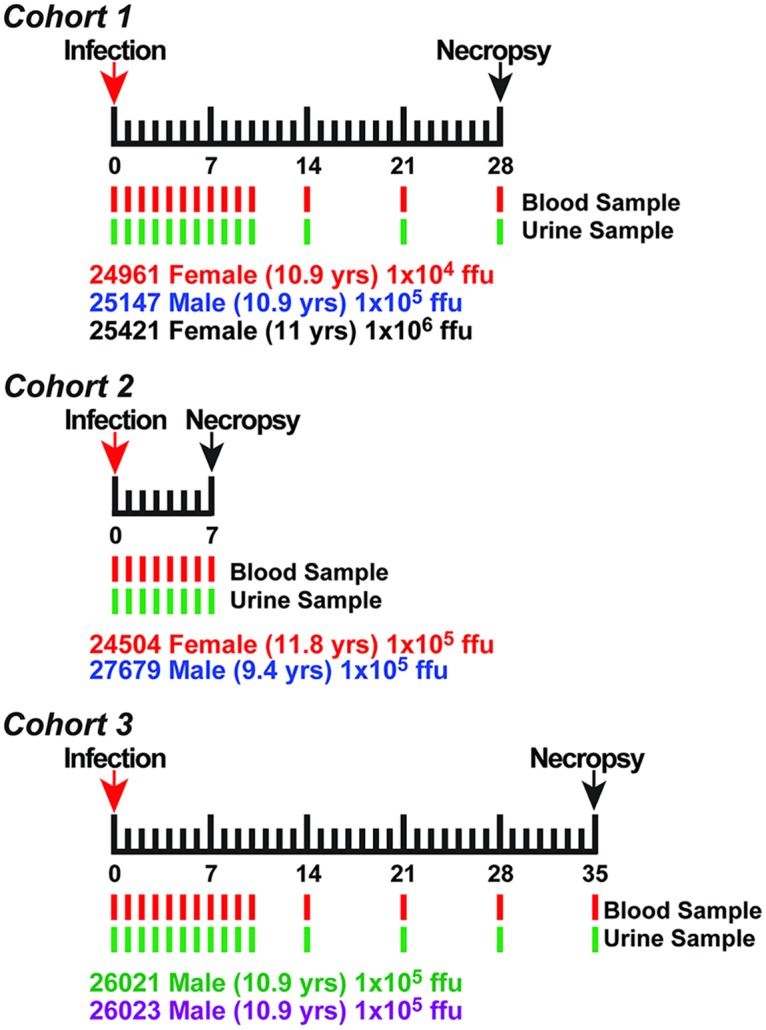
Cohort design and timeline used for ZIKV infection of rhesus macaques. Graphical depiction of the cohorts and timeline used for the infection of Rhesus monkeys with ZIKV. Blood and urine were collected daily for the first 10 days post infection and then at 14, 21, 28, and 35 dpi. Cohorts 1 and 2 and 3 were taken to necropsy at 28, 7, and 35 dpi, respectively. Cohort 1 was used to examine ZIKV dose effect and viral persistence. Cohorts 2 and 3 was used to determine viral tissue tropism during acute infection as well as at later times after resolution of serum viremia. Monkey identification number, age and sex as well as the infectious dose of ZIKV used to infect the animals are listed for each cohort.

Seven Indian-origin RMs (3 females and 4 males) were divided into three cohorts (Fig 1 contains a description of the animals within each cohort). Cohort 1 was infected subcutaneously with a total of 1x10^4^, 1x10^5^, or 1x10^6^ focus forming units (ffu) of ZIKV diluted in 1ml of PBS and delivered by ten 100μl injections into the hands and arms bilaterally. These doses are comparable to the 1x10^5^ pfu median dose of the flavivirus West Nile virus (WNV) previously determined to be delivered by the bite of infected *Culex* spp mosquitoes [27]. Cohorts 2 and 3 were similarly infected with 1x10^5^ ffu. Peripheral blood and urine (collected in the cage pan) samples were obtained at 0, 1, 2, 3, 4, 5, 6, 7, 8, 9, 10, 14, 21, 28 and 35 dpi. Peripheral blood mononuclear cells (PBMCs) and plasma samples were separated by centrifugation over lymphocyte separation medium. PBMCs were analyzed for immune cell phenotype and frequency by flow cytometry. Plasma was assessed for viral loads by qRT-PCR and the levels of cytokines by Luminex multiplex-bead based assay, as described below. Urine was assessed for viral RNA by qRT-PCR and for infectious virus by co-culture on C6/36 cells and focus-forming assays using Vero cells. Cohort 1 was euthanized at 28 dpi, Cohort 2 animals at 7 dpi, and Cohort 3 at 35 dpi. Samples of tissues (joints, muscles, organs, brain, spinal cord, peripheral nerves, glands, and lymph nodes) and biological fluids (cerebral spinal fluid, blood, and urine) were collected and stored in RNAlater, Trizol (RNA isolation), medium (virus isolation) as well as fixed and embedded in paraffin.

### Enzyme-Linked Immunosorbent Assay (ELISA)

Plasma anti-ZIKV antibody concentrations were measured by end point dilution ELISA. For this assay, high-binding polystyrene 96-well plates (Corning) were coated with PBS containing a 1:1000 dilution of 4x10^8^ ffu/ml stock of purified ZIKV particle preparations The plates were incubated overnight at 4°C and then blocked with PBS containing 2% milk and 0.05% Tween (ELISA-Block) for 1 hr at room temperature. Plates were washed with 0.05% Tween-PBS (ELISA-Wash) and incubated with two-fold dilutions of RM plasma in ELISA-Block starting at a dilution of 1:50. The plate was incubated at room temperature for 2 hrs. Plates were washed several times with ELISA-Wash and then incubated with secondary anti-monkey IgM or IgG (Rockland, Inc.) conjugated with horseradish peroxidase for 30 mins. Plates were washed with ELISA-Wash and bound secondary antibody was detected using the OPD substrate (Life Technologies) followed by HCl stop the assay. The plates were read within 10 minutes using a Synergy HTX Microplate Reader (BioTek) at 490nm. Endpoint titers of ZIKV binding antibodies were determined using a Log/Log transformation method and the results were analyzed and graphed using GraphPad Prism v6 software.

### Anti-ZIKV neutralization assay

Neutralization assays were used to measure the concentration of serum that can neutralize 50% of a fixed number of ZIKV (50% Plaque Reduction Neutralization Test (PRNT50)). Sera from infected RM were serially diluted 4-fold from starting dilutions of 1:10 and following dilution were mixed with an equal volume of ZIKV (30–50 PFU) for final serum dilutions ranging from 1:20 to 1:5120. Sera and virus were incubated for 1hr at 37°C. The mixtures were added to individual wells of 24-well plates seeded with Vero cells at 90% confluence for 1hr at 37°C on a rocker and then overlaid with 1% methylcellulose in OPTI-MEM (Gibco). Plates were incubated for 3 days at which time the cells were fixed and counterstained with methyl blue. Plaques were visualized and counted using a light box. Raw counts were entered into GraphPad Prism v. 6.0, converted to a percent of mock neutralized input virus and PRNT50 values were calculated utilizing the sigmoid dose-response curve fitting function with upper and lower limits of 100 and 0, respectively.

### One-Step qRT-PCR analysis

RNA from tissue samples, blood, urine, and cerebrospinal fluid (CSF) was isolated using TRIzol (Invitrogen) according to the manufacturer’s protocol. ZIKV RNA levels were measured by a one-step quantitative real time reverse transcription polymerase chain reaction assay (qRT-PCR) using TaqMan One-Step RT-PCR Master Mix (Applied Biosystems). 250ng total RNA from tissue samples or 1/10^th^ volume of RNA isolated from 100 μl of liquid samples was used in each reaction. Primers and probes were as follows: Forward: 5’-TGCTCCCACCACTTCAACAA (ZIKV PRVABC59 genome sequence nucleotides 9797–9816); Reverse: 5’-GGCAGGGAACCACAATGG; (complement of nucleotides 9840–9857); and TaqMan probe: 5’ Fam-TCCATCTCAAGGACGG-MGB (nucleotides 9819–9834). Forward and reverse primers were used at 250 nM in the reaction, and the probe at 200 nM. Validation of the qRT-PCR assay is shown in S1 Fig. For RNA standards, RNA was isolated from purified, titered stock of ZIKV (PRVABC59). RNA yield was quantified by spectrometry and the data was used to calculate genomes/μl. Focus-forming units (ffu)/ μl was calculated based on titer of stock. ZIKV RNA was serially diluted 1:10 into Vero cell RNA (25 ng/μl) and amplified in triplicate using primers and conditions described above.

### ZIKV isolation from tissues

Tissues were homogenized in 1ml of DMEM cell culture medium containing 5% FBS and PSG plus approximately 250μl of SiLiBeads using a bead beater (Precellys 24 homogenizer, Bertin Technologies), and cellular debris were pelleted by centrifugation (5,000 × *g* for 2 min). A 50μl or 500μl sample of the clarified lysate was applied to one well of a 6-well plate of C6/36 cells for seven days. Supernatant titers from these cultures were transferred to Vero cells, incubated at 37°C for an additional 5 d and assayed for the presence of infectious virus by indirect immunofluorescent staining using mAb 4G2 and an anti-mouse IgG Alexa-488 conjugated secondary antibody. This method proved must sensitive in isolation of infectious virus, as compared to initially culturing tissue samples with Vero cells.

### Phenotypic analysis of peripheral blood mononuclear cells

Flow cytometry was used to quantify the immune cell phenotype as well as the level of cellular proliferation and activation for peripheral blood mononuclear cells (PBMCs) isolated at the time points defined above. The panel of antibodies used for the analysis of innate immune cells consisted of HLA-DR, CD14, CD11c, CD123, CD20, CD3, CD8, CD16, and CD169. To differentiate between monocyte/macrophages, DCs, and NK cells the following gating strategy was utilized: monocyte/macrophages (CD3^-^CD20^-^CD14^+^HLA-DR^+^), myeloid DCs (CD3^-^CD20^-^CD14^-^HLA-DR^+^CD11c^+^), plasmacytoid DCs (CD3^-^CD20^-^CD14^-^HLA-DR^+^CD123^+^), other DCs (CD3^-^CD20^-^CD14^-^HLA-DR^+^CD123^-^CD11c^-^), and natural killer (NK) cells (CD3^-^CD20^-^CD8^+^CD16^+^). The percentage of activated cells (CD169^+^) within each subset was calculated as a representation of the cellular activation profile [28]. T cells were analyzed with the following panel of antibodies directed against CD4, CD8β, CD95, CD28, CD127 and for intracellular levels of Ki67 (proliferation marker). The T cell subset was identified as CD4^+^ or CD8^+^ and within the CD4^+^ and CD8^+^ T cell subsets, the naïve (CD28^+^CD95^-^), central memory (CD28^+^CD95^+^), and effector memory (CD28^-^CD95^+^) subsets are displayed. B cells were analyzed using the following antibodies: CD3, CD20, CD27, and IgD to delineate naïve (CD3^-^CD20^+^CD27^-^IgD^+^), memory (CD3^-^CD20^+^CD27^-^IgD^-^) and marginal-zone like B cells (CD3^-^CD20^+^CD27^+^IgD^+^) as well as Ki67 to identify proliferating cells. The percentage of proliferating (Ki67^+^) B and T cells within each subset was calculated as well as for granzyme B (activation marker). The gating strategies and definition of the different cellular subsets were performed as previously described [18]. Phenotyping was performed using an LSRII instrument (BD bioscience) and the data was analyzed with FlowJo Software (TreeStar).

### Serum cytokine and blood chemistry assays

Monkey Cytokine Magnetic 29-plex Panel (Luminex Platform Kit from Invitrogen) was used to quantify cytokine and chemokine expression in blood plasma and CSF samples. According to the manufacturer’s instructions, antibody-conjugated polystyrene magnetic beads were plated onto a 96-well plate and washed with buffer. Beads were incubated with a 7-point standard curve along with 25μl of rhesus monkey plasma or CSF plus 25μl of blocking buffer for 2h. Beads were washed with wash buffer and labeled with the biotinylated detector antibody for 1hr. Beads were washed and then incubated with Streptavidin conjugated to R-Phycoerythrin for 30 minutes and washed. After final wash, cytokines were identified and quantified using a Luminex 200 Detection system (Luminex). Statistical analysis was performed using Sidak’s multiple comparison tests and data was graphed using GraphPad Prism v6 software.

Whole blood chemistry analysis was performed on a VetScan VS2 system (Abaxis, Union City CA) using the 14-analyte Mammalian Comprehensive Diagnostic Profile Panel (#500–0038) according to the manufacturer instructions.

### Histological analysis of ZIKV disease

Complete necropsies were performed and tissues were collected for microscopic examination. Tissues were fixed in 10% buffered formalin, embedded in paraffin, sectioned at 5μm and stained with hematoxylin and eosin.

### ZIKV in situ hybridization

*In situ* hybridization studies were performed on formalin fixed paraffin-embedded (FFPE) tissue sections of 5μm using two different Zika-specific commercial RNAscope Target Probes (Advanced Cell Diagnostics, Hayward, CA; catalog #464531 and #463781) complementary to sequences 866–1763 and 1550–2456, respectively. Pretreatment, hybridization and detection techniques were performed according to manufacturer’s instructions. In the absence of control specimens of Zika virus infected cells/tissues, FFPE brain tissue from mice infected with West Nile Virus [29] were used as positive controls. The target probe #463781 detected WNV infected brain tissue while the probe #464531 did not. Both probes detected ZIKV in the test specimens. Tissue sections were counterstained with hematoxylin. As a negative control, a probe specific for Influenza A virus (ACD catalog #313241 was used to probe contiguous sections of Zika positive tissues.

### Magnetic-Activated Cell Separation (MACS) of splenocytes and axillary lymph node cells

Spleen and lymph node tissues were dissected at necropsy to produce single cell suspensions by first pushing the tissues through a wire mesh filter followed by extensive washing, and lysis of red blood cells. These cell preparations were put through a 70μm filter and counted prior to being frozen in RPMI containing 50% fetal calf serum plus 10% DMSO in liquid nitrogen. For MACS the cells were thawed, pelleted by low speed centrifugation (1,500 rpm for 10 minutes) and resuspended in MACS buffer at a concentration of approximately 2x10^7^ cells/mL. Prior to magnetic separation, cells were passed through a 70*μ*M filter, to remove cell clumps. The cells were then divided into two aliquots of 1x10^7^ cells/mL per tissue type. One aliquot was used to obtain pure populations of CD14^+^ macrophages and CD3^+^ T cells and the second to obtain pure populations of CD20^+^ B cells and CD1c^+^ dendritic cells. All cell types were isolated using a two-step magnetic isolation method with RM-specific reagents (MACS, Miltenyi Biotec, Germany).

For isolation of T cells and macrophages, approximately 1x10^7^ cells were incubated with magnetic beads coated with anti-CD14 (Miltenyi Biotech) for 15 min at 4°C. The cells were then washed and resuspended in 500*μ*L MACS buffer before being loaded onto a LD magnetic separation column. After sample loading the column was washed with 2mL of MACS buffer. The magnetically labeled CD14^+^ macrophages retained on the column were eluted by removing the column from the magnetic field and flushing with MACS buffer using the provided plunger. A portion of the eluted cells were saved for purity analysis via flow cytometry, the remaining eluted cells were pelleted and resuspended in Trizol reagent. The CD14-depleted cells in the flow-through were pelleted, resuspended in MACS buffer and incubated with magnetic beads coated with anti-CD3-biotin antibody (Miltenyi Biotech) for 10 min at 4°C, followed by subsequent incubation with anti-biotin MicroBeads (Miltenyi Biotech) for 15 min. The cells were then washed and resuspended in 500*μ*L MACS buffer before being loaded onto a MS magnetic separation column for positive selection. Labeled CD3^+^ T cells, retained on the column, were flushed out of the column with 1mL MACS buffer with the provided plunger in the absence of a magnetic field. Eluted cells were analyzed for purity and vRNA as above.

For isolation of CD20^+^ B cells and CD1c^+^ DCs, the total cell mixtures were first incubated with a FcR blocking reagent (MACS) and magnetic beads coated with anti-CD1c-PE (MACS) for 5 mins at 4°C followed by a 15 mins incubation with magnetic bead coated with anti-CD20 (MACS). Cells were then washed, resuspended in 500*μ*L MACS buffer, and loaded onto a LD magnetic separation column. The CD20^+^ B cells retained on the column were eluted with MACS buffer as described above, and a portion was analyzed for purity via flow cytometry. The remaining eluted cells were pelleted and resuspended in Trizol reagent. To positively select for CD1c^+^ DCs the B cell-depleted flow-through fraction was pelleted, resuspended in MACS buffer, and then incubated with anti-PE Microbeads (MACS) for 15 mins at 4°C. The cells were then washed and resuspended in 500*μ*L MACS buffer before being loaded onto a MS magnetic separation column. The CD1c^+^ DCs retained on the column were eluted and analyzed as above.

### NF-κB reporter assay

Luciferase assays were performed as previously described [30]. Briefly, the firefly luciferase (LUC) open reading frame downstream of an NF-κB promoter element was transduced via lentivector (Qiagen) into RM fibroblasts whose functional lifespan was extended through the stable introduction of human telomerase. Cells were grown, infected, and treated in 96 well plates as indicated. After adding Steady Glo lysis and luciferin reagent (Promega) luminescence was read on a BioTek Synergy plate reader.

## Results

### Infection and clinical characterization of RM cohorts

Three cohorts of RMs were used for this study (Fig 1). The first cohort consisted of two adult females and one adult male. Animals were infected with 1x10^4^, 1x10^5^, or 1x10^6^ focus forming units (ffu) of ZIKV (PRVABC59). The infectious dose was divided over 10 subcutaneous injections over bilateral hands and arms. Blood and urine were sampled daily through 10 dpi, as well as on 14, 21, and 28 dpi. Euthanasia was performed at 28 dpi and tissues collected at necropsy for analysis of viral loads. A second cohort, consisting of two adult RM (one male, one female) was infected with 1x10^5^ ffu, followed by daily sampling of blood and urine through 7 dpi, at which time animals were euthanized as above. The third cohort, consisting of two adult male RM, was infected with 1x10^5^ ffu followed by daily sampling of blood and urine through 35 dpi. All animals developed a transient fever, rash on the arms and upper torso, as well as lymphadenopathy of their axillary lymph nodes. Additionally, 3 of 7 animals developed conjunctivitis lasting 3–5 days. None of the infected animals experienced weight loss or signs of clinical disease other than those described above. Analysis of blood chemistry revealed no significant changes following ZIKV infection (S2 Fig).

### ZIKV viral loads and tissue tropism

**Fig 2:**
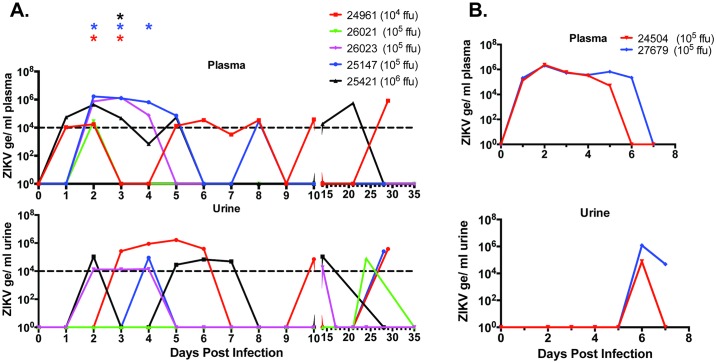
Viral loads in plasma and urine from ZIKV-infected rhesus macaques. One-step qRT-PCR was used to measure ZIKV RNA loads in the plasma (top panels) and urine (bottom panels) from each animal at indicated days pi and represented as copies per milliliter of fluid. (A) Cohort 1 and 3: Animal 24961(1x10^4^ ffu)-red lines; 25147 (1x10^5^ ffu)-blue lines; 25421 (1x10^6^ ffu)-black lines; 26021 (1x10^5^ ffu)-green lines; 26023 (1x10^5^ ffu)-purple lines. (B) Cohort 2: Animal 24504 (1x10^5^ ffu)-red lines; and Animal 27679 (1x10^5^ ffu)-blue lines. 1/10^th^ of total RNA extracted from 100 μl plasma or urine was used in each reaction. Approximate limit of detection at 1e4 genomes/ml is based on a detection limit of ~100 genomes in each reaction (S1 Fig) as indicated by the dotted line. Asterisks indicate infectious virus co-cultured from plasma harvested on day shown.

All infected animals developed plasma viremia as detected by RT-qPCR of viral genomes that typically peaked at 2 dpi and was detectable out to 5–7 dpi (Fig 2A and 2B). We were unable to titer virus directly from plasma samples. However, infectious virus, detected by co-culture of plasma with C6/36 cells, was observed in indicated cases between 2 to 4 dpi (Fig 2A, stars). Viral RNA in the urine was detected from 3–10 dpi with peak levels at 5 dpi (Fig 2A and 2B). We detected viral RNA positive urine samples outside of the initial 3–10 dpi window, which is consistent with other reports of ZIKV infections of NHP [22]. This finding indicates that ZIKV infection in NHP is dynamic and remains persistent.

**Fig 3:**
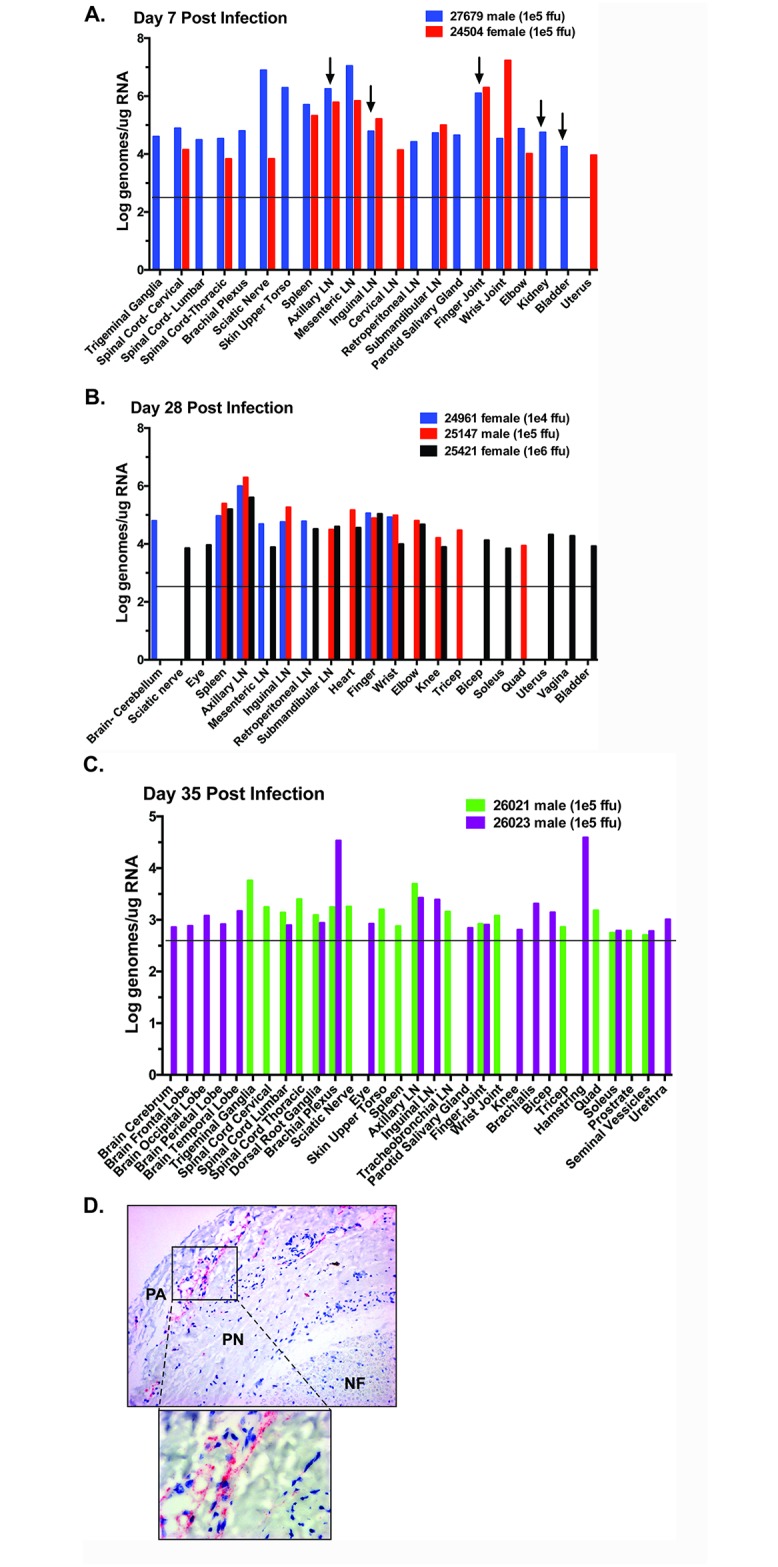
Viral loads in the tissues following necropsy of ZIKV-infected rhesus macaques. One-step qRT-PCR was used to measure ZIKV RNA loads in the tissues of animals in Cohort 2 (A), Cohort 1 (B), and Cohort 3 (C). Total RNA was generated using the Trizol method on precleared samples following bead beating. Approximately 80 different tissues were assessed for the presence of viral RNA. Shown are the tissues with positive detection in at least one of the animals per cohort. Arrows indicate samples in which virus was successfully co-cultured from tissue homogenate. Approximate limit of detection at 1e4 genomes/ml is based on a detection limit of ~100 genomes in each reaction (S1 Fig) as indicated by the horizontal line. (D) Paraffin sections of sciatic nerve cut in cross section were hybridized with ZIKV specific chromogenic probe (red) and counterstained with hematoxylin (blue). Nerve fibers (NF) show a normal distribution within the endoneurium surrounded by perineurium (PN) and perineurial adventia (PA). Hybridization for ZIKV was robust but limited to the PA region. Original magnification was 50X.

Following euthanasia and necropsy, RNA was isolated from individual tissues and the viral genomes were quantified by qRT-PCR (viral loads of positive tissues in Fig 3A and 3B; complete list of tissues samples in S1 Table). At 7 dpi (cohort 2, 1x10^5^ ffu), viral RNA was detected in multiple tissues: lymphoid tissue—including lymph nodes distributed throughout the body as well as the spleen; joints—most prominently joints near the site of inoculation but in some instances more distal joint tissue as well; peripheral nervous tissue, specifically the sciatic nerve, brachial plexus and trigeminal ganglion. Additionally, viral RNA was found associated with the spinal cord (cervical, lumbar and thoracic), but not in CSF or in the brain, at this time point. This may indicate neurological tropism, but an inability to effect retrograde transport of infectious virus into the CNS or that infection of the CNS requires additional time. Viral RNA was detected in the kidney and bladder of the male animal (27679), although not in the testes or prostate. Viral RNA was found in the uterus of the female (24504).

We were able to co-culture infectious ZIKV in C6/36 insect cells from homogenates of the axillary and inguinal lymph nodes, finger joints, kidney and bladder derived from the male monkey (27679) (Fig 3A, black arrows). Together these data indicate that ZIKV quickly disseminates to many tissues throughout the body including lymph nodes/spleen, peripheral nerves, and skin as well as the genital/urinary tract.

At 28 dpi, (cohort 1, inoculated with 1x10^4^ 1x10^5^ and 1x10^6^ ffu) viral RNA was still detected in both lymphoid and joint tissues in all animals (Fig 3B). In general, vRNA tissue distribution was greatest for the 1x10^5^ and 1x10^6^ ffu infected animals. The axillary (draining) lymph nodes and spleen showed the highest level of viral RNA in all three animals, while other lymph nodes were positive for at least 2 of 3 animals. Joint tissues close to the site of inoculation (wrist and finger) were also positive in all three animals 28 dpi. Additional joints, muscles of the arms and legs, and heart were positive for ZIKV RNA in subsets of animals. In animal 25421 (female, 1x10^6^ ffu) viral RNA was detected in the reproductive tissues (uterus and vagina) suggesting that the virus can infect these tissues and persist there for at least 4 weeks post infection. This finding may have important implications for viral transmission and fetal infections during pregnancy. Viral RNA was also detected in the sciatic nerve and eyes from this subject. Interestingly, ZIKV RNA was detected in the cerebellum of animal 24561 (female, 1x10^4^ ffu), indicating penetration to the CNS. Co-culture of homogenates from tissues collected at 28 dpi with C6/36 cells did not amplify infectious virus.

At 35 dpi, (cohort 3, inoculated with 1x10^5^ ffu) positive viral RNA detection occurred in neuronal tissues, lymph nodes, and joint/muscle tissues (Fig 3C). Animal 26023 displayed extensive neuronal tissue involvement with viral RNA detected in the occipital and parietal lobes of the brain, lumbar region of the spinal cord, dorsal root ganglia, brachial plexus, and eye. In Animal 26021, ZIKV RNA was not detected in the brain but was present in the trigeminal ganglia, as well as cervical, lumbar and thoracic regions of the spinal cord and peripheral nerves (brachial plexus and sciatic nerve). Interestingly, *in situ* hybridization on cross sections of sciatic nerve using ZIKV-specific chromogenic probes detected robust virus RNA levels in the perineurial adventitial space from animal 26023 (Fig 3D). Virus was not detected in the nerve fibers. Both animals had viral RNA in their axillary lymph nodes. These results combined with the viral detection data from the day 28 animals confirm the long-term persistence of ZIKV RNA in neuronal, lymph node and joint/muscle tissues.

Histologic examination of sections taken from tissues of infected RM found few specific abnormalities, although several areas of inflammation were observed (S3 Fig). An uncharacteristic prostatitis characterized by interstitial neutrophilic and lymphoplasmacytic cellular infiltrates and glandular microabscesses were noted 7dpi in animal 27679 infected with 1x10^5^ ffu (S3A Fig). Minimal perivascular lymphocytic or lymphoplasmacytic inflammatory cell infiltrates were present in sections of skin from the upper torso affected with a rash for both animals examined 7dpi (S3B Fig). Viral RNA was also detected in this area of skin in this animal. Similarly, variable perivascular inflammatory infiltrates composed of lymphocytes, eosinophils and plasma cells were observed in the joints and muscles of animal 24504 (S3C & S3D Fig). Focal lymphohistiocytic inflammation was associated with a meningeal vessel in the cerebrum of the high dose (1x10^6^ ffu) animal 25421 at 28 dpi suggestive of an ongoing infection of the brain vasculature (S3E Fig). Animal 25147 (28 d pi, 1x10^5^ ffu) had focal lymphocytic infiltration of the dorsal root ganglion of the cervical spinal cord (S3F Fig). The lack of correlation of detection of viral RNA with sites of inflammation in the prostate, brain, and DRG may indicate highly focal areas of infection, or clearance of virus prior to resolution of inflammation.

**Fig 4:**
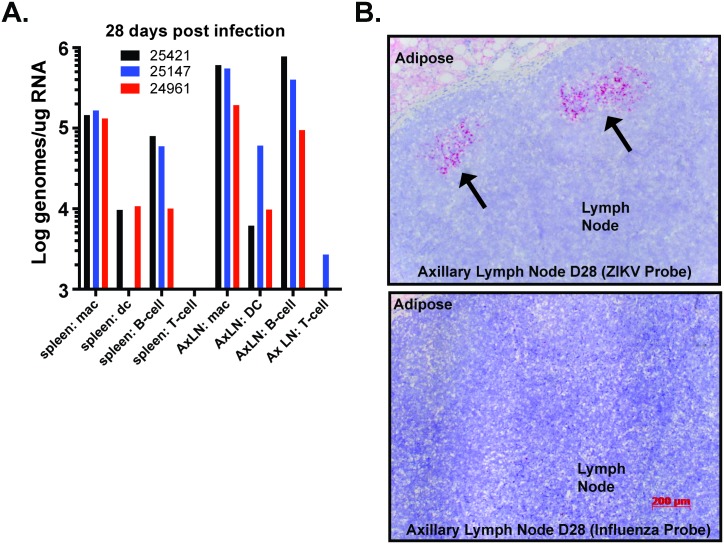
Infected cell types in spleen and axillary lymph nodes of ZIKV-infected rhesus macaques. Cell subpopulations were isolated by positive selection magnetic bead separation from lymphocytes isolated from the spleen and axillary lymph nodes at 28 dpi. For macrophage and T cell isolation, CD14-microbeads were used to isolate macrophages from total spleen and axillary lymph node lymphocytes, and then anti-CD3 was used to isolate T cells from macrophage depleted flow-through. For B cell and DC isolation, total splenocytes or axillary lymph node lymphocytes were first positively selected for CD20^+^ B cells and the depleted fraction was bound to CD1c microbeads to isolate DCs. To increase purity of the isolated cell populations all positively selected samples were eluted after primary selection and then re-bound to a second fresh column. Depiction of isolated cell populations as characterized by flow cytometry is shown in S4 Fig. Total RNA was isolated from the positively selected cell fractions and quantified. One-step qRT-PCR was used to measure ZIKV RNA loads in each of the cell fractions isolated from animals at 28dpi (A). Viral RNA loads were highest in the macrophage and B cell fractions, with consistently less vRNA detected in DCs and rarely in the T cells. (B) Serial paraffin sections of axillary lymph node tissue from animal #25421 were hybridized with ZIKV specific chromogenic probe (red) or Influenza specific chromogenic probe (red) and counterstained with hematoxylin (blue) showed strong positive staining for ZIKV in the lymphoid follicles. Original magnification was 50X.

In order to determine which cell types within lymphoid tissues were positive for viral RNA, we sorted macrophage, dendritic cell, B-cells and T-cells from the splenocytes and axillary lymphocytes by positive magnetic bead selection (S4 Fig). RNA was isolated from each cell population and ZIKV RNA quantified by qRT-PCR. As shown, at 28 dpi RNA is primarily found in the macrophage and B cell subsets with reduced levels in DC subsets but rarely present in the T cell fractions (Fig 4A). *In situ* hybridization with ZIKV- and Influenza-specific probes detected ZIKV but not Flu RNA in multiple axillary lymph node follicles from animal #25421 (Fig 4B), confirming the presence of the ZIKV RNA in the macrophage, B cell and DC rich regions of the germinal center. Overall, this data indicates that ZIKV spreads to multiple tissue types and the infection of many of these tissues persists in macrophages, as well as other cell types for at least 4–5 weeks post infection.

### Immune activation following ZIKV infection

**Fig 5:**
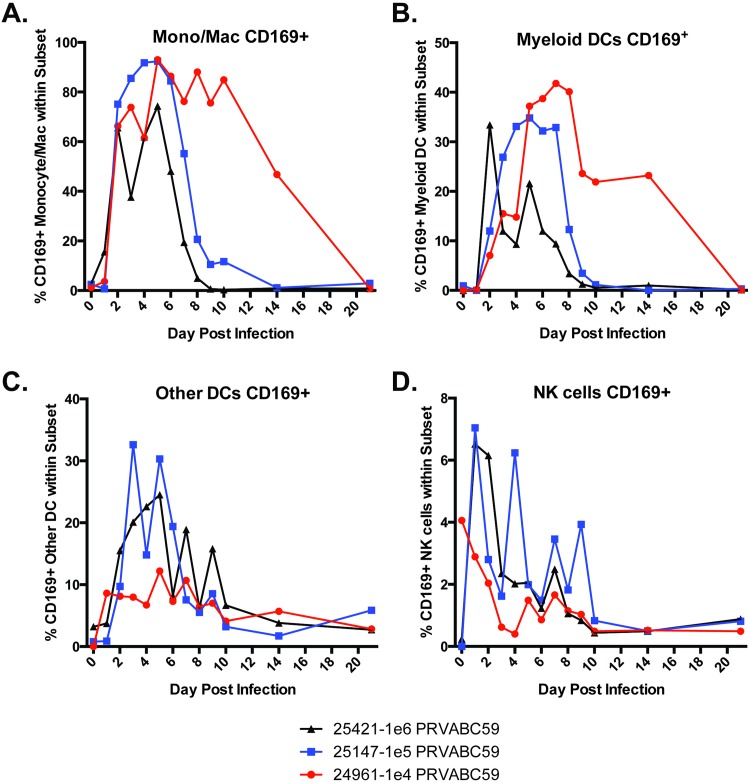
ZIKV-infection induces robust innate immune cell activation. Total peripheral blood mononuclear cells from all time points were stained with fluoroflore-conjugated antibodies directed against the cellular markers CD3, CD8, CD11c, CD14, CD16, CD169 and HLA-DR in order to assess changes in the activation of A) monocyte/macrophages; B) myeloid dendritic cells; C) other dendritic cells; and D) NK cells. Multi-color flow cytometry was used to visualize the stained cells. The percentage of activated cells (CD169^+^) was calculated using FlowJo and the data was graphed in GraphPad Prism v6 software.

We performed a detailed phenotypic analysis of immune cell subsets by flow cytometry to characterize activation of innate immune cells (monocyte/macrophage/DC/NK cells) as well as adaptive immune cell proliferative responses (T and B cells). We also characterized cytokines and antibodies present in the sera of infected RM. Within 1–2 days pi, all of the animals showed innate immune cell activation, as demonstrated by the presence of CD169^+^ staining (Fig 5). RM 24961 (1x10^4^ ffu) displayed a more protracted innate immune response, compared to 25421 (1x10^6^ ffu), 25147, 26021 and 26023 (1x10^5^ ffu). While all animals showed an increase in CD169^+^ monocytes and DCs at 2–4 dpi, the number of activated cells waned between day 8–10 pi in animals infected with 1x10^5^ or 1x10^6^ ffu, while the number of activated cells in the animal infected with 1x10^4^ ffu did not return to baseline levels until 14–21 dpi.

**Fig 6:**
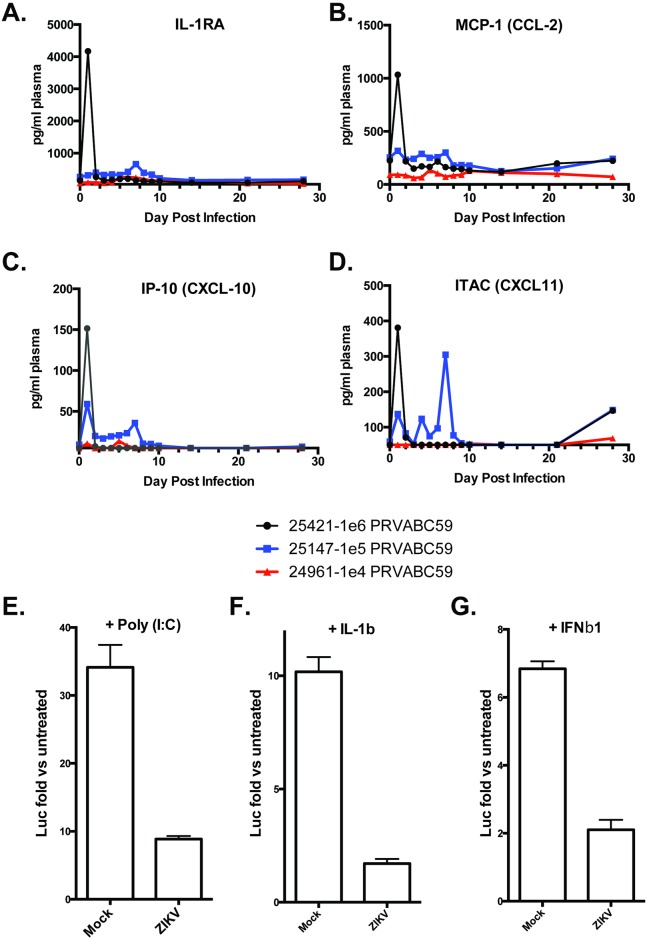
Rhesus cytokine and chemokine production in response to ZIKV infection and block of NF-kB signaling in Rhesus fibroblasts. A 29-plex-cytokine/chemokine/growth factor magnetic bead assay was performed on plasma from rhesus monkeys at all time points post infection. Cytokine analysis revealed changes in only A) IL-RA; B) MCP-1-CCL2; C) IP-10-CXCL10; and D) ITAC-CXCL11. Reporter assay showing induction of NF-κB-dependent (E, F) or interferon stimulated response element (ISRE)-dependent (G) LUC expression in fibroblasts infected for 56h with ZIKV at MOI = 5ffu/cell. Luminescence was measured 8h after treatment with 60μg/mL poly(I:C) (E) 100ng/mL human IL-1β (F) or 5,000 units/ml IFNβ1 (G). Values displayed are average fold changes (three replicates) of stimulated versus untreated cells ±SD.

Cytokine expression in the plasma largely did not change following infection. However, expression of 4 cytokines (IL-1RA, MCP-1-CCL2, IP-10-CXCL10, and I-TAC-CXCL11) was induced over background levels in the plasma (Fig 6A–6D). Expression of these cytokines was elevated within the first several days post infection but returned to baseline levels by 10 dpi. Low levels of cytokine activation *in vivo* may be an indirect effect of routine ketamine treatment [31] or a result of direct inhibition by ZIKV of innate immune pathways that direct synthesis and secretion of pro-inflammatory cytokines. To examine the latter possibility we employed a reporter assay for which the readout is luciferase expression that responds to NF-κB or JAK/STAT pathway (type I IFN) activation [32,33]. As shown in Fig 6E and 6F, rhesus fibroblasts infected with ZIKV for 48h at 5 FFU/cell showed significantly diminished LUC signal relative to uninfected cells following treatment with either poly(I:C) or human IL-1β. These represent distinct NF-κB-terminal signaling pathways with poly(I:C) induction resulting from activation of the TLR3 pattern recognition receptor and TRIF adaptor protein as well as IL-1β triggering the IL1 receptor and associated MyD88 adaptor protein. Similarly, activation of JAK/STAT signaling by IFNβ1 treatment was also repressed by ZIKV infection (Fig 6G). These results agree with previous observations that ZIKV infection promotes degradation of STAT2 and subsequent inhibition of type I IFN signaling [34]. As such we hypothesize that ZIKV exhibits an inhibitory phenotype that operates downstream of the convergence of these pathways, likely targeting activation of NF-κB itself but delimiting the mechanisms associated with this point will require further experimentation.

**Fig 7:**
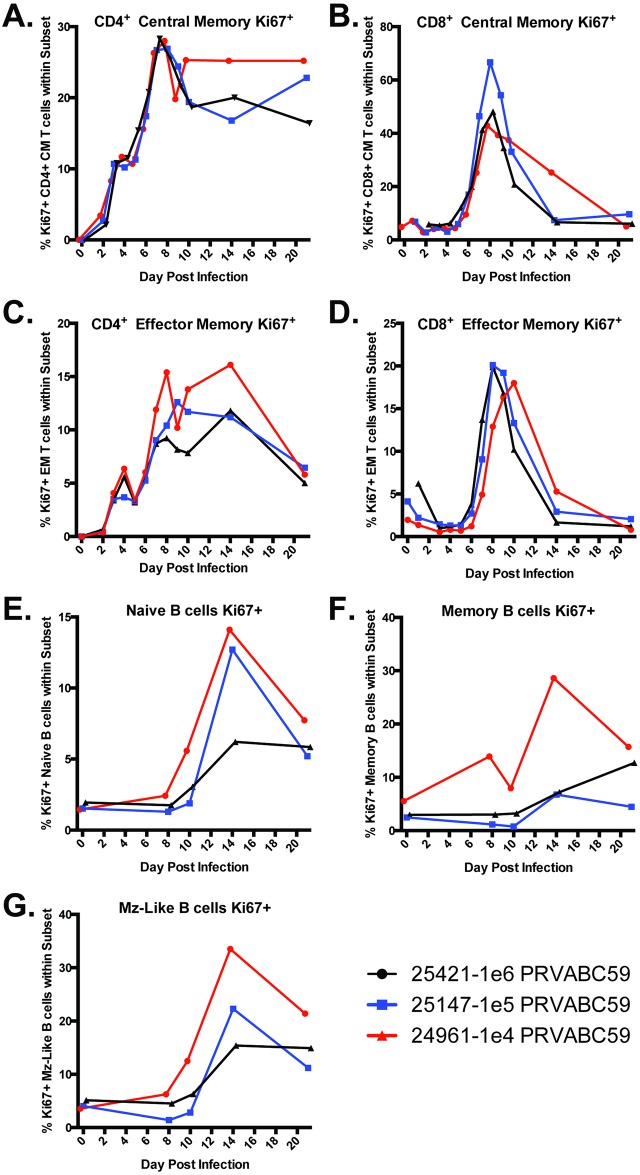
Rhesus adaptive immune cell proliferative responses following ZIKV infection. Total peripheral blood mononuclear cells were analyzed by flow cytometry for the presence of T and B cell proliferative responses following infection. T cells were identified by staining with antibodies directed against the cellular markers CD3, CD4, CD8β, CD95, CD28, CD127 and for intracellular levels of Ki67 (proliferation marker) to assess changes the proliferation of A) CD4^+^ central memory T cells; B) CD8^+^ central memory T cells; C) CD4^+^ effector memory T cells; and D) CD8^+^ effector memory T cells. B cells were stained with antibodies directed against CD3, CD20, CD27, IgD and HLA-DR as well as Ki67 in order to compare the proliferative responses in E) naïve B cell; F) memory B cells; and G) marginal-zone like B cells. The percentage of actively proliferating cells (Ki67^+^) was calculated using FlowJo and the data was graphed in GraphPad Prism v6 software.

Proliferating CD4^+^ and CD8^+^ T-cells were present in all infected animals by 6–8 dpi. CD8^+^ T cell proliferative responses (Ki67^+^ cells) were evident at 6 dpi, maximal at 8–9 dpi and returned to background levels by 14 dpi (Fig 7B and 7D). Both central memory and effector memory CD4^+^ T cell proliferative responses were maximal at 7 dpi (Fig 7A and 7C) but took longer to return to baseline levels compared to the CD8^+^ T cells. Consistent with these findings, Granzyme B expression in naïve and central memory CD4^+^ and CD8^+^ T cells peaked between 7 and 10 days post infection (S5 Fig).

B cell proliferative burst responses were maximal at 14 dpi (Fig 7E–7G). Interestingly, when comparing the cohort 1 animals, the B-cell proliferative responses in RM 24961 (1x10^4^ ffu) were observed slightly earlier than in RMs 25147 (1x10^5^ ffu) and 25421 (1x10^6^ ffu) and represented a greater percentage of cells within each subset. Proliferating T-cells also appeared for a longer time post infection in this animal (Fig 7A–7D).

**Fig 8:**
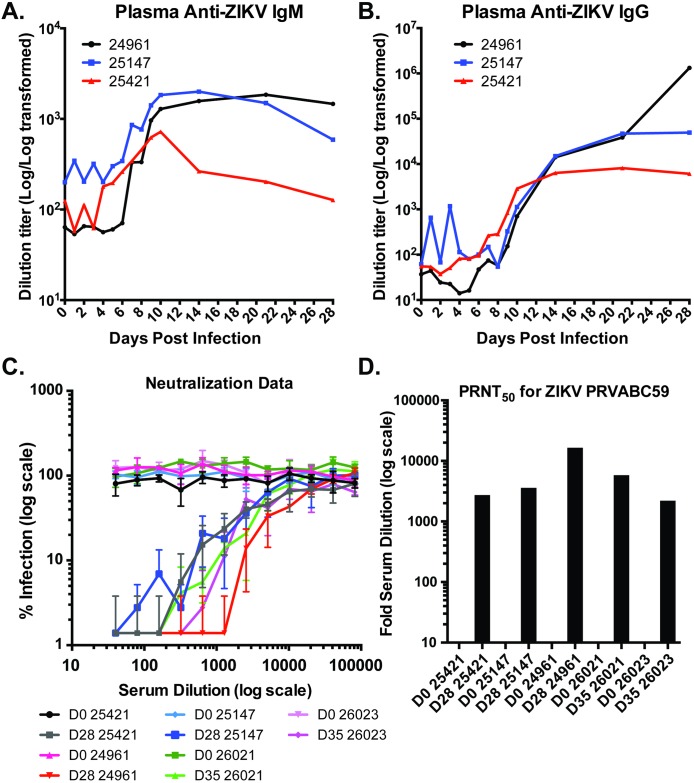
Detection of anti-ZIKV antibody responses in Rhesus plasma. Rhesus macaques infected with ZIKV were analyzed for the presence of antibodies directed against ZIKV-PRABC59 by ELISA using whole virus as capture antigen with an HRP-conjugated anti-Rhesus IgM **(A)** or IgG **(B)** secondary antibody. **C.** Sera from indicated animals obtained pre-infection (d0) or at terminal bleed (d28 or 35 pi) were tested for neutralizing activity via plaque reduction neutralization titer (PRNT) assay. **D.** Fold dilution giving 50% reduction in infectious titer for each serum sample.

ZIKV virion-reactive IgM and IgG antibodies in sera were quantified by ELISA. Levels of anti-ZIKV IgM became detectable between 7–10 dpi and were maintained through 28 dpi in 2 of 3 animals, while one animal (#25421) showed reduced titers after 10 dpi. (Fig 8A). IgG levels increased beginning between d8 to d14 pi and plateaued around 21 dpi (animals #25147 and #25421) or continued to increase in the low dose animal (#24961). Western blotting of ZIKV infected cell lysates revealed that antibody responses targeted at least two proteins that were 38 and 55 kDa, respectively, consistent with viral proteins NS1 and E (S6 Fig), which elicit antibody responses during ZIKV infection of humans and are also major antibody targets during other flavivirus infections [35,36]. The neutralizing capacity of the ZIKV-directed antibodies was quantitated, and robust neutralizing antibody responses were detected at 28 or 35 dpi (Fig 8C and 8D) in all animals, regardless of the infectious dose.

## Discussion

In 1947, the Zika virus was originally isolated in Uganda from a febrile RM used as a sentinel in a study of yellow fever virus transmission. Given this initial finding, we hypothesized that ZIKV infection of RM could be used as a model of viral replication, pathogenesis, and immune response. Our results demonstrate that the 2015 Puerto Rico ZIKV strain productively infects the RM, characterized by clinical symptoms comparable to that described in human infection, such as fever, rash, and conjunctivitis. The RM further develops viremia, viruria, widespread tissue infection and a robust adaptive immune response. These data are generally in agreement with recently published studies of RM infected with a 2013 isolate from French Polynesia [22].

Because ZIKV has shown a breadth of tissue tropism not seen in other human flavivirus infections, we sought to characterize distribution of ZIKV in RMs as broadly as possible. As such, our study significantly advances what is known about the tissue tropism of ZIKV on 7, 28 and 35 dpi, a key feature of infection not examined by previous RM studies at these time points. Analysis of viral genome load in tissues revealed a tropism for lymphoid, joint and peripheral nerve tissue. The apparent persistence of viral RNA in various tissues after resolution of primary viremia is not unique to ZIKV. Persistence of flaviviruses in the infected host after cessation of viremia and recovery from clinical symptoms (if any) has been observed in several instances. In humans who have been infected with WNV, prolonged viruria (in several cases >6 years) is sometimes observed [37]. This viral persistence may correlate with long-term neurological and renal sequelae [38,39]. Models of WNV persistence in RM, mice, and hamsters indicate that virus can persist for months in tissues of the CNS, kidney, and lymphoid organs, and the presence of virus also correlates with long term neurological sequelae and motor neuron loss in hamsters [21,40–43]. Herein, we demonstrate that ZIKV RNA was detected in peripheral nerves (5 out of 7 RM) and spinal cord (4 out of 7 RM) and viral RNA can persist in these tissues for up to 5 weeks post infection, which could lead to long-term neuropathology. Indeed, post-recovery complications from ZIKV infection, primarily GBS or other neurologic manifestations, have been documented in the French Polynesian and subsequent South American outbreaks. GBS is characterized by the presence of certain autoreactive antibodies and immune cells and is often associated with previous infection, including viral infection. It is unknown how either acute or chronic ZIKV infection might contribute to GBS or other symptoms, and warrants further study.

Additionally, viral RNA was detected in tissues of the male and female reproductive tracts, which may have implications for the association of ZIKV infection of pregnant women with aberrant fetal development and sexual transmission. These findings also correspond to a similar observation of ZIKV RNA in the genital tract of a human female [44], and may also suggest a mechanism of a recently described instance of female to male sexual transmission [45]. In male RMs, we were unable to detect viral RNA in the testes, which is, perhaps, surprising given the reports of male to female ZIKV sexual transmission. However, we were able to detect viral RNA in the prostate and seminal vesicles, which may represent a potential reservoir and mode of sexual transmission. In addition, the presence of virus in the bladder and urine suggests virus seeding into the semen in the urethra may also be a possible route of transmission. Further intensive study with regard to sexual transmission is clearly warranted. We also note that the challenge route during sexual transmission may affect the biological outcomes to ZIKV infection including pathogenesis. While our studies were designed to mimic mosquito transmission, further studies to elucidate the effect of route of infection on disease are possible in RM. Viral RNA detected in the vagina and uterus of infected females may also be relevant to the association between ZIKV infection during pregnancy and microcephaly/fetal abnormalities. Interestingly, early results from one such study observed prolonged viremia in females infected during the first trimester of pregnancy, leading the authors to speculate that the fetus may be the source of this virus, or that the placenta may serve as a primary reservoir of ongoing ZIKV infection [22]. Our results suggest that other tissues within the infected dam, such as the female reproductive tract, lymphoid or joint tissue may also be considered as a potential source of persistent virus, given that pregnancy is associated with partial immune suppression, the pregnant animal maybe unable to completely clear viral infection. Again, much more intensive analysis of how ZIKV affects pregnancy in RM will be informative.

Several recent studies have examined ZIKV infection *in vivo* using various mouse models. These include ZIKV challenge of mice deficient in type I or type I and II interferon (IFN) receptors, in which infection is lethal. Additionally, some strains of immunocompetent mice also appear susceptible to ZIKV infection with regard to displaying detectable viremia and susceptibility of fetuses to developmental defects, although the mechanisms underlying the outcomes in this model are unclear. In type I IFN receptor deficient mice (IFNAR-/- or A129) viral tropism appears broader than we observe in the infected RM. Notably, high viral loads were observed in the brain and testes of these mice, tissues that in the RM, had undetectable or minimal levels of viral RNA. These results suggest that restriction of viral tropism *in vivo* may be due to the action innate immune factors. Therefore, robust and highly relevant animal models of disease are critical to advancing our understanding of ZIKV disease pathogenesis, immune responses, and potential vaccination and antiviral strategies and these will include both mouse and nonhuman primate models. Although it is a flavivirus, ZIKV clinical and virologic features are distinct and strikingly different from other flavivirus infections, challenging pre-existing assumptions about how this virus behaves in an intact host. Results presented here establish the outstanding potential of the RM ZIKV model for dissecting many of these unique features—specifically effective infectious dose, tissue tropism, fluid compartment infection, and chronicity of infection—in a host that both naturally develops disease and has a fully intact immune system. The magnitude and ongoing expansion of the current ZIKV outbreak calls for rapid initiation of more comprehensive studies to further validate and expand this RM model as well as begin to explore specific *in vivo* questions that are critical to controlling the ZIKV epidemic.

## Supporting information

S1 Table Complete list of tissues examined for ZIKV RNA. +: ZIKV RNA detected (see Fig 3 for values); -: ZIKV RNA below limit of detection; LN: lymph node.
(PDF)

S1 Fig Validation of taqman qRT-PCR quantitation of ZIKV RNA. RNA was isolated from purified titered stock of ZIKV (PRV- ABC59). RNA yield was quantified by spectrometry and used to calculate genomes/ μl. Focus-forming units (ffu)/ μl was calculated based on titer of stock. ZIKV RNA was serially diluted 1:10 into Vero cell RNA (25 ng/μl) and amplified in triplicate using primers and conditions described in methods. Amplification cycle threshold (CT) is plotted against total viral genomes (A) or total ffu (b).
(TIF)

S2 Fig Blood chemistry for RM infected with ZIKV. Serum chemistry analysis was performed at 0, 1, 2, 3, 4, 5, 6, 7, 8, 9 days post infection.
(TIF)

S3 Fig Histological images of tissues from ZIKV-infected animals at 7 and 28 dpi. Formalin-fixed tissue sections were stained with heamtoxylin and eosin. Shown a representative images of stained sections of (A) prostatitis in animal #27679 at 7 dpi; (B) perivascular lymphocytic infiltration in rash area of upper thorax skin of animal #27679 at 7 dpi; (C) perivascular lymphocytic infiltration in finger joint from animal #24504 at 7 dpi; (D) perivascular lymphocytic infiltration in right triceps muscle of animal #24504; (E) lymphocytic infiltration of the cerebral meninges of animal #24521 at 28 dpi; and (F) lymphocytic infiltration present in the dorsal root ganglion of animal #25147 at 28dpi. Arrows denote areas of inflammation.
(TIF)

S4 Fig Infected cell types in spleen and axillary lymph nodes of ZIKV-infected rhesus macaques. In order to identify the origin of the infected cells present in lymph tissues, cell subpopulations were isolated by positive selection magnetic bead separation from lymphocytes isolated from the spleen and axillary lymph nodes at 28 dpi. CD14-microbeads were used to isolate macrophages from total spleen and axillary lymph node lymphocytes, and then anti-CD3 was used to isolate T cells from macrophage depleted flow-through. B cells were first positively selected for CD20+ and the depleted fraction was bound to CD1c microbeads to isolate DCs. All positively selected samples were eluted after primary selection and then re-bound to a second fresh column. Characterization of isolated cell populations by flow cytometry is shown.
(TIF)

S5 Fig ZIKV infection results in upregulation of Ki67 and granzyme B in CD4 and CD8 T cells. The mean frequencies of Ki67 positive (Panels A and B) and granzyme B positive (Panels C and D) T cells within Naive, central memory (CM) and effector memory (EM) subsets of CD4 (Panels A and C) and CD8 (Panels B and D) in PBMC from Animals 26021 and 26023.
(TIF)

S6 Fig Antibodies from infected RM primarily recognize ZIKV E and NS1 proteins. Vero cells were infected with ZIKV at MOI = 0.5 or 5 ffu/cell. At 48 h pi, cell lysates were collected, and proteins from infected cell lysates as well as uninfected Vero cells (lanes “-”) were resolved by SDS-PAGE. Proteins were probed by western blotting using plasma from indicated animals diluted 1:200, and anti-monkey Ig 2° Ab conjugated to HRP (Rockland Immunochemicals). Anti-flavivirus E mAb 4G2 was used to visualize E expression as well (right panel). Arrows indicate expected positions of E (upper arrow) and NS1 (lower arrow).
(TIF)
