# Correction: Zika Virus infection of rhesus macaques leads to viral persistence in multiple tissues

**DOI:** 10.1371/journal.ppat.1006317

**Published:** 2017-04-05

**Authors:** Alec J. Hirsch, Jessica L. Smith, Nicole N. Haese, Rebecca M. Broeckel, Christopher J. Parkins, Craig Kreklywich, Victor R. DeFilippis, Michael Denton, Patricia P. Smith, William B. Messer, Lois M. A. Colgin, Rebecca M. Ducore, Peta L. Grigsby, Jon D. Hennebold, Tonya Swanson, Alfred W. Legasse, Michael K. Axthelm, Rhonda MacAllister, Clayton A. Wiley, Jay A. Nelson, Daniel N. Streblow

The following information is missing from the Funding section: Oregon National Primate Research Center Pilot Project awarded through the Division of Pathobiology and Immunology and the Division of Reproductive and Developmental Sciences (grant P51-OD011092). The correct funding information is as follows: This work was supported by Oregon National Primate Research Center Pilot Project awarded through the Division of Pathobiology and Immunology and the Division of Reproductive and Developmental Sciences (ONPRC core grant P51-OD011092) as well as the National Institutes of Health (Grant U42 OD010426). The funders had no role in study design, data collection and analysis, decision to publish, or preparation of the manuscript.
