# Supplementary material for: Zika Virus infection of rhesus macaques leads to viral persistence in multiple tissues
Source: PLoS Pathog. 2017 Mar 9;13(3):e1006219. doi: 10.1371/journal.ppat.1006219 (PMC5344528; doi:10.1371/journal.ppat.1006219)
Supplement: S1 Table — +: ZIKV RNA detected (see Fig 3 for values); -: ZIKV RNA below limit of detection; LN: lymph node. (PDF) [file ppat.1006219.s001.pdf]

	7 dpi	
	27679	24504
Tissue	male (1e5 ffu)	female, (1e5 ffu)
Ankle	-	+
Axillary LN	+	+
Bicep	-	-
Bladder	+	-
Brachia	-	-
Brachial plexus	+	-
Brain- Cerebellum	-	-
Brain- Frontal	-	-
Brain- Hind	-	-
Cervical LN	-	+
Cervix	-	-
Elbow	+	+
Eye	-	-
Finger	+	+
Hamstring	-	-
Heart	-	-
Inguinal LN	+	+
Kidney	+	-
Knee	-	-
Liver	-	-
Lung	-	-
Mesenteric LN	+	+
Ovary	-	-
Parotid Salivary gland	+	-
Prostate	-	-
Quadriceps	+	-
Retroperitoneal LN	+	-
Sciatic Nerve	+	+
Skin	+	-
Soleus	+	-
Spinal Cord- Cervical	+	+
Spinal Cord- Lumbar	+	-
Spinal Cord-Thoracic	+	+
Spleen	+	+
Submandibular gland	-	-
Submandibular LN	+	+
Testes	-	-
Toe	+	-
Tricep	+	-
Trigeminal ganglion	+	-
Uterus	-	+
Vagina	-	-
Wrist	+	+

	28 dpi		
	24961	25147	25421
Tissue	female (1e4 ffu)	male (1e5 ffu)	female (1e6 ffu)
Adrenal gland	-	-	-
Ankle	-	-	-
Axillary LN	+	+	+
Bicep	-	-	+
Bladder	-	-	+
Brachia	-	-	-
Brachial plexus	-	-	-
Brain- cerebellum	+	-	-
Brain- frontal	-	-	-
Brain- hind	-	-	-
Cervical LN	-	-	-
Cervix	-	-	-
Elbow	-	+	+
Eye	-	-	+
Finger	+	+	+
Hamstring	-	-	-
Heart	-	+	+
Inguinal LN	+	+	-
Kidney	+	-	-
Knee	-	+	+
Liver	+	-	-
Lung	-	+	-
Mesenteric LN	+	-	+
Ovary	-	-	-
Parotid Salivary gland	-	-	-
Prostate	-	-	-
Quadriceps	-	+	-
Retroperitoneal LN	+	-	+
Salivary gland LN	-	+	+
Sciatic nerve	-	-	+
Seminal vesicles	-	-	-
Skin	-	-	-
Soleus	-	-	+
Spinal Cord- Cervical	-	-	-
spinal cord- lumbar	-	-	-
Spinal Cord- Thoracic	-	-	-
Spleen	+	+	+
Testes	-	-	-
Submandibular gland	-	-	-
Toe	-	-	-
Tricep	-	+	-
Trigeminal ganglion	-	-	-
Uterus	-	-	+
Vagina	-	-	+
Wrist	+	+	+

	35 dpi	
	27679	24504
Tissue	male (1e5 ffu)	male (1e5 ffu)
Ankle	-	-
Axillary LN	+	+
Bicep	+	-
Bladder	-	-
Brachia	+	-
Brachial plexus	+	+
Brain- cerebellum	+	-
Brain- frontal	+	-
Brain-occipital	+	-
Brain- parietal	+	-
Brain- temporal	+	-
Cervical LN	-	-
Dorsal root ganglion	+	+
Elbow	-	-
Epididymus-body	-	-
Epididymus-head	-	-
Epididymus-tail	-	-
Eye	+	-
Finger	+	+
Hanstring	+	-
Heart	-	-
Inguinal LN	+	-
Kidney	-	-
Knee	+	-
Liver	-	-
Lung	-	-
Mesenteric LN	-	-
Parotid Salivary gland	+	-
Prostate	-	+
Quadriceps	-	+
Retroperitoneal LN	-	-
Salivary gland LN	-	-
Sciatic nerve	-	+
Spinal Cord- Cervical	-	+
spinal cord- lumbar	+	+
Spinal Cord- Thoracic	-	+
Seminal vesicle	+	+
Skin	-	+
Soleus	+	+
Spleen	-	+
Submandibular gland	-	-
Tracheobronchial LN	-	+
Testes	-	-
Toe	-	-
Tricep	-	+
Trigeminal ganglia	-	+
Urethra	+	-
Vas deferens	-	-
Wrist	-	+

Supplemental Table 1: Complete list of tissues examined for ZIKV RNA. +: ZIKV RNA detected (see Fig. 3 for values); -: ZIKV RNA below limit of detection; LN: lymph node
