# Supplementary figures and images for: Zika Virus infection of rhesus macaques leads to viral persistence in multiple tissues
Source: PLoS Pathog. 2017 Mar 9;13(3):e1006219. doi: 10.1371/journal.ppat.1006219 (PMC5344528; doi:10.1371/journal.ppat.1006219)

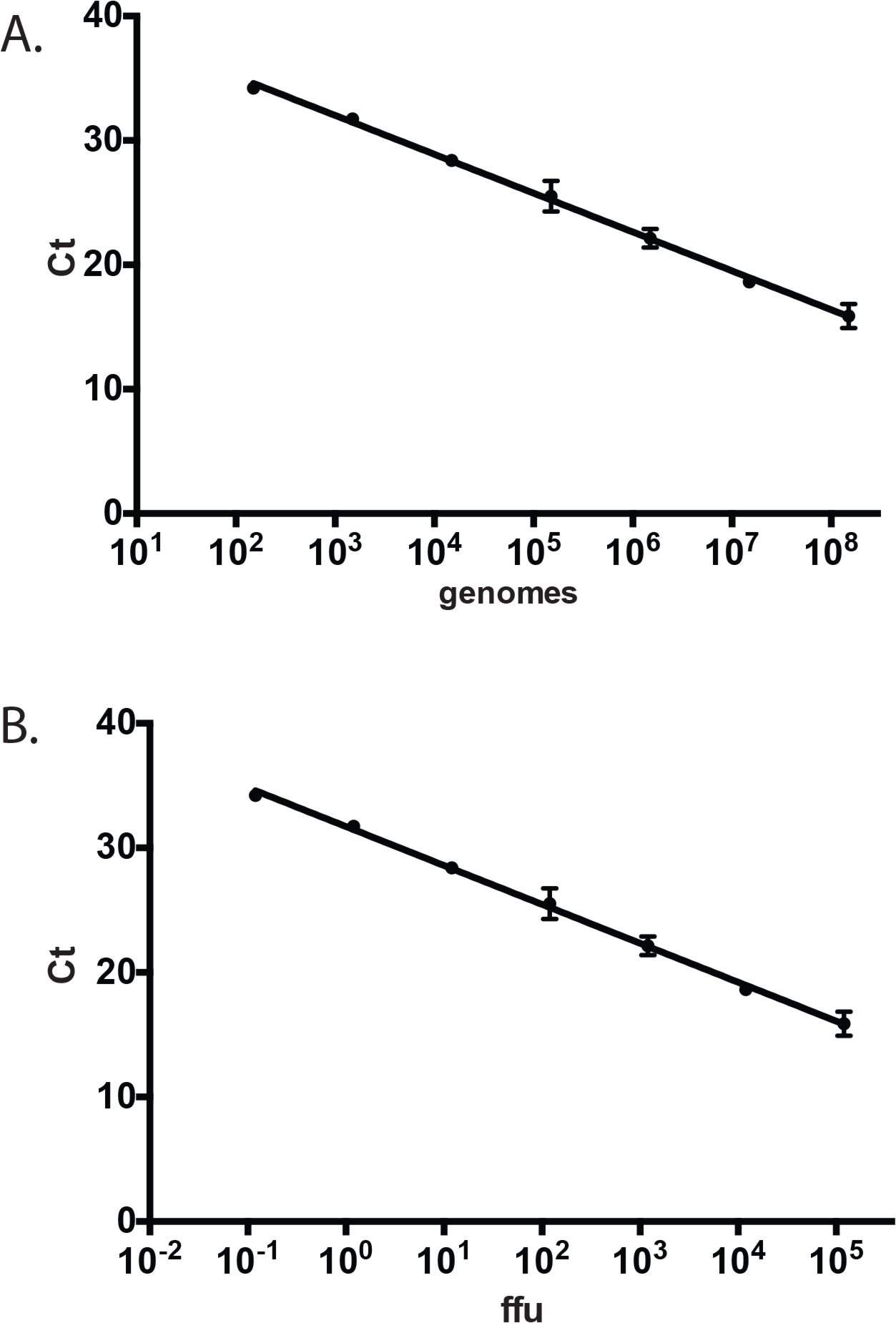

Supplement: S1 Fig — RNA was isolated from purified titered stock of ZIKV (PRV- ABC59). RNA yield was quantified by spectrometry and used to calculate genomes/ μl. Focus-forming units (ffu)/ μl was calculated based on titer of stock. ZIKV RNA was serially diluted 1:10 into Vero cell RNA (25 ng/μl) and amplified in triplicate using primers and conditions described in methods. Amplification cycle threshold (CT) is plotted against total viral genomes (A) or total ffu (b). (TIF) [file ppat.1006219.s002.tif]

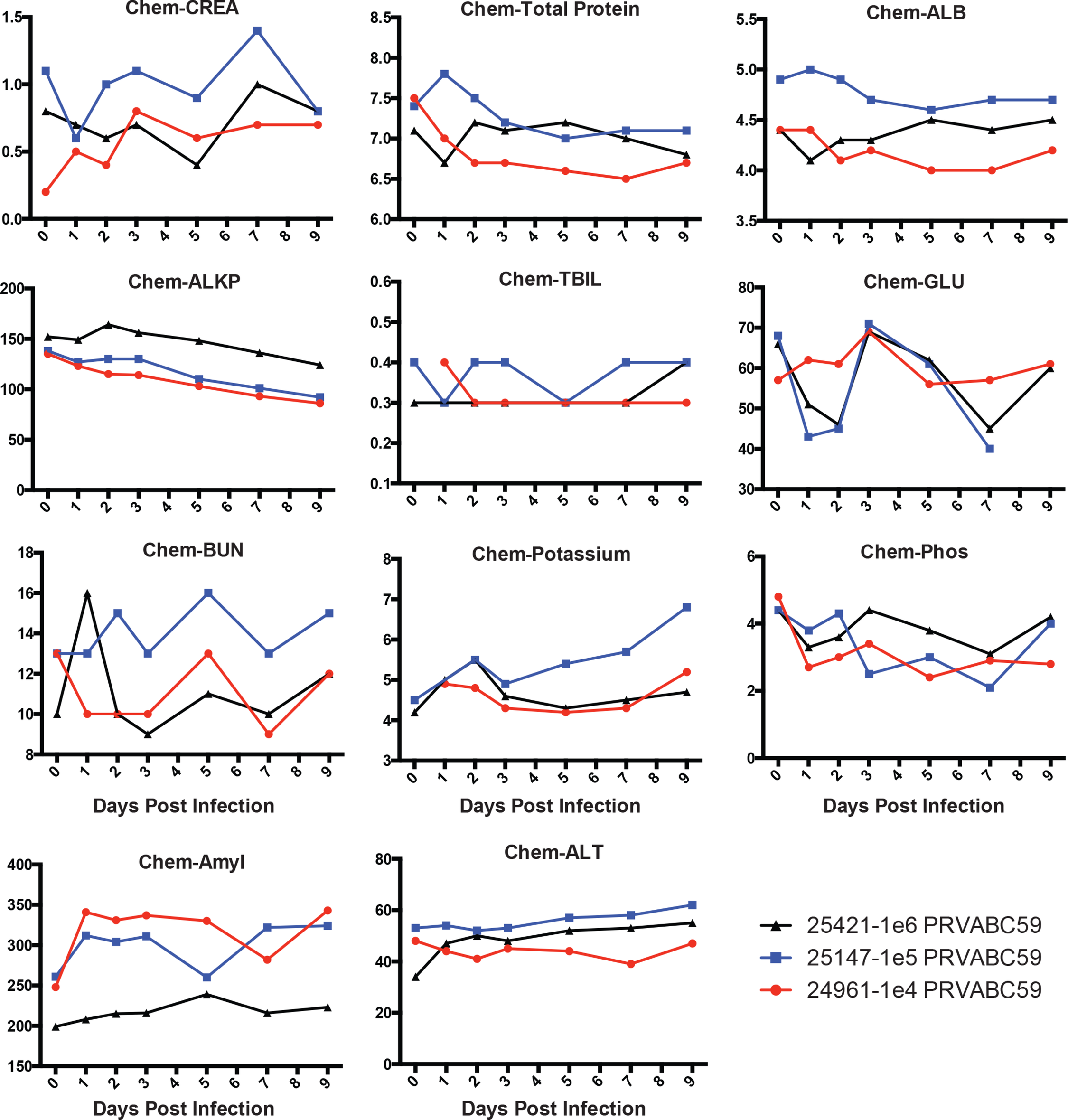

Supplement: S2 Fig — Serum chemistry analysis was performed at 0, 1, 2, 3, 4, 5, 6, 7, 8, 9 days post infection. (TIF) [file ppat.1006219.s003.tif]

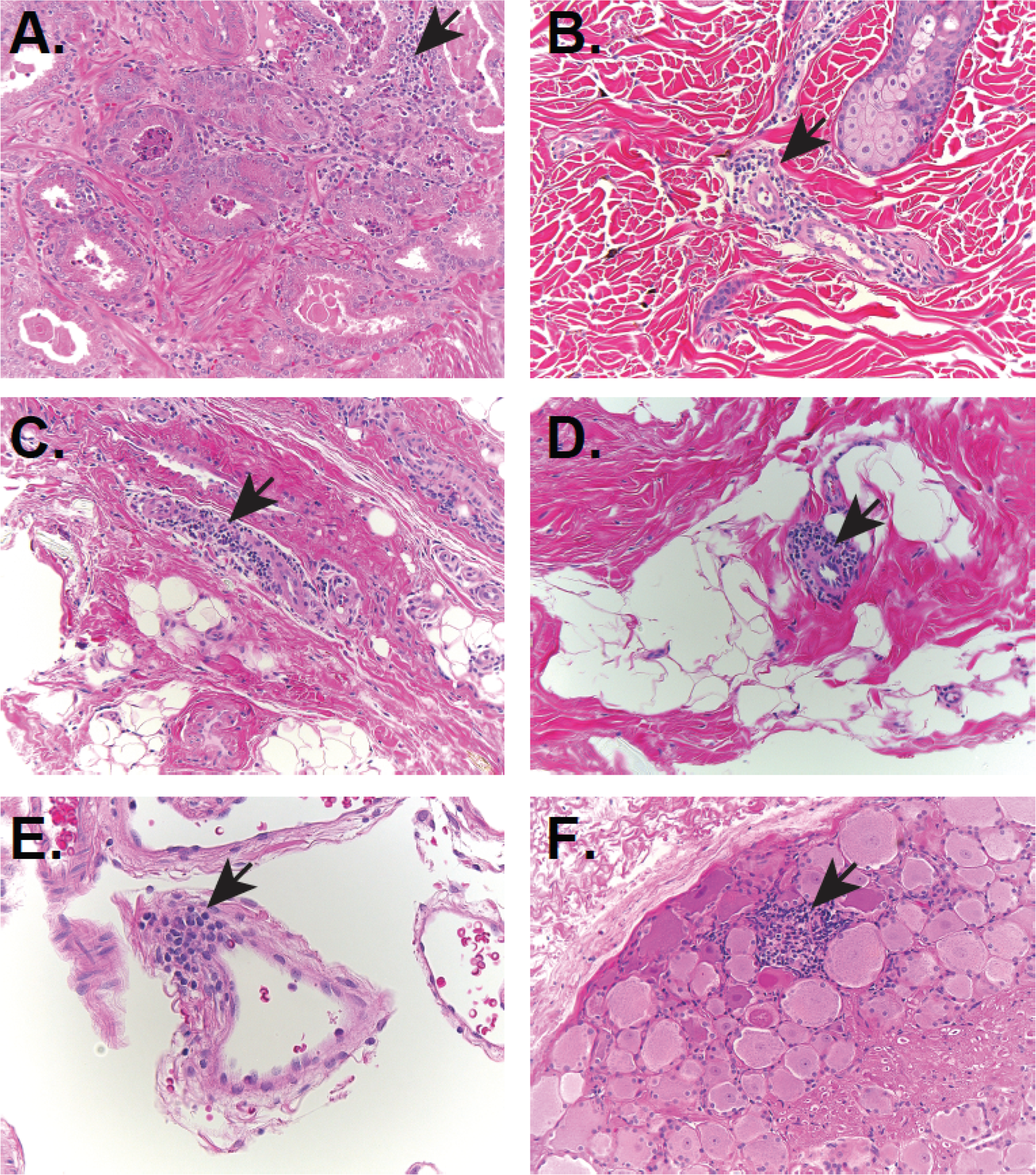

Supplement: S3 Fig — Formalin-fixed tissue sections were stained with heamtoxylin and eosin. Shown a representative images of stained sections of (A) prostatitis in animal #27679 at 7 dpi; (B) perivascular lymphocytic infiltration in rash area of upper thorax skin of animal #27679 at 7 dpi; (C) perivascular lymphocytic infiltration in finger joint from animal #24504 at 7 dpi; (D) perivascular lymphocytic infiltration in right triceps muscle of animal #24504; (E) lymphocytic infiltration of the cerebral meninges of animal #24521 at 28 dpi; and (F) lymphocytic infiltration present in the dorsal root ganglion of animal #25147 at 28dpi. Arrows denote areas of inflammation. (TIF) [file ppat.1006219.s004.tif]

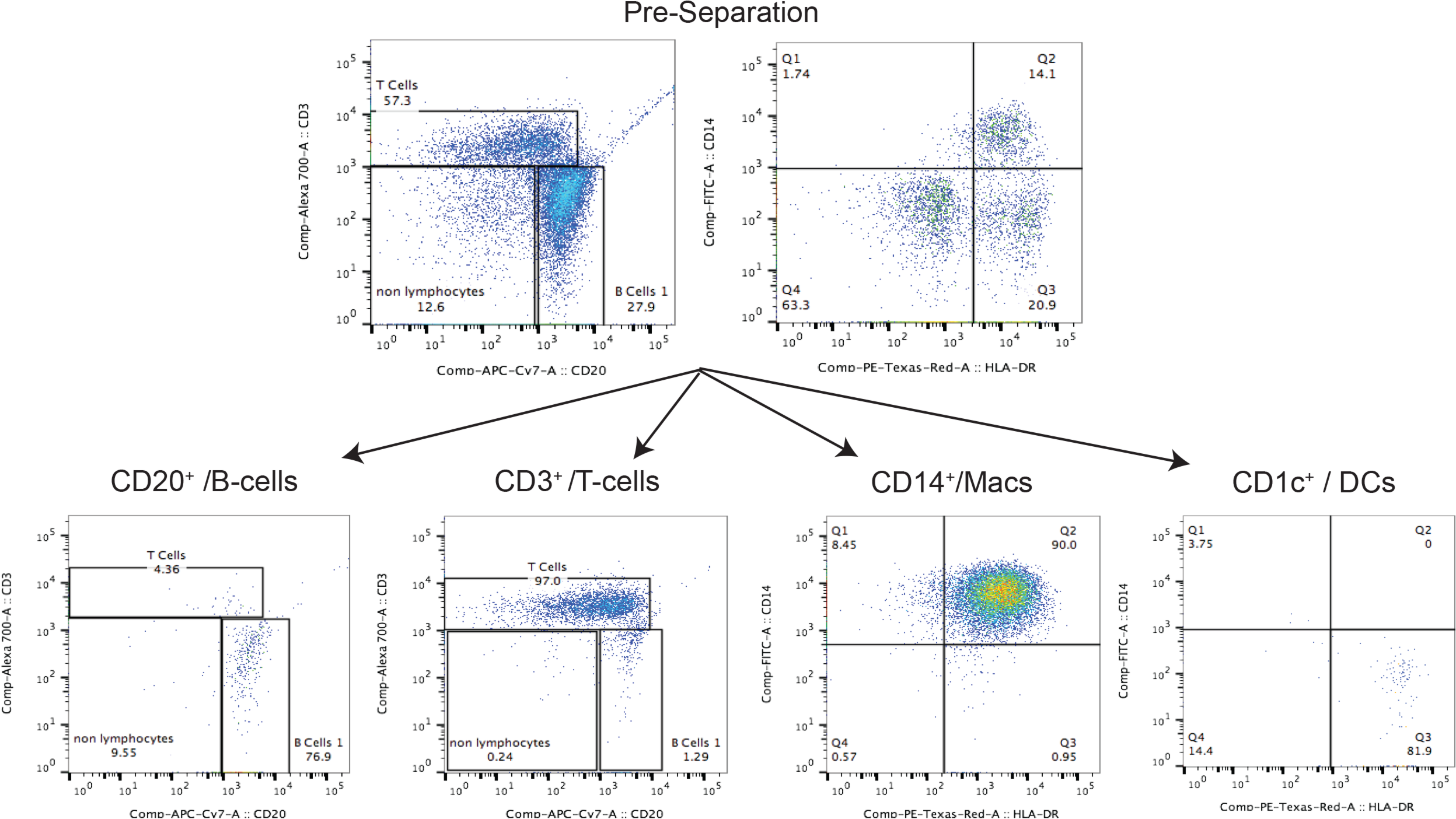

Supplement: S4 Fig — In order to identify the origin of the infected cells present in lymph tissues, cell subpopulations were isolated by positive selection magnetic bead separation from lymphocytes isolated from the spleen and axillary lymph nodes at 28 dpi. CD14-microbeads were used to isolate macrophages from total spleen and axillary lymph node lymphocytes, and then anti-CD3 was used to isolate T cells from macrophage depleted flow-through. B cells were first positively selected for CD20+ and the depleted fraction was bound to CD1c microbeads to isolate DCs. All positively selected samples were eluted after primary selection and then re-bound to a second fresh column. Characterization of isolated cell populations by flow cytometry is shown. (TIF) [file ppat.1006219.s005.tif]

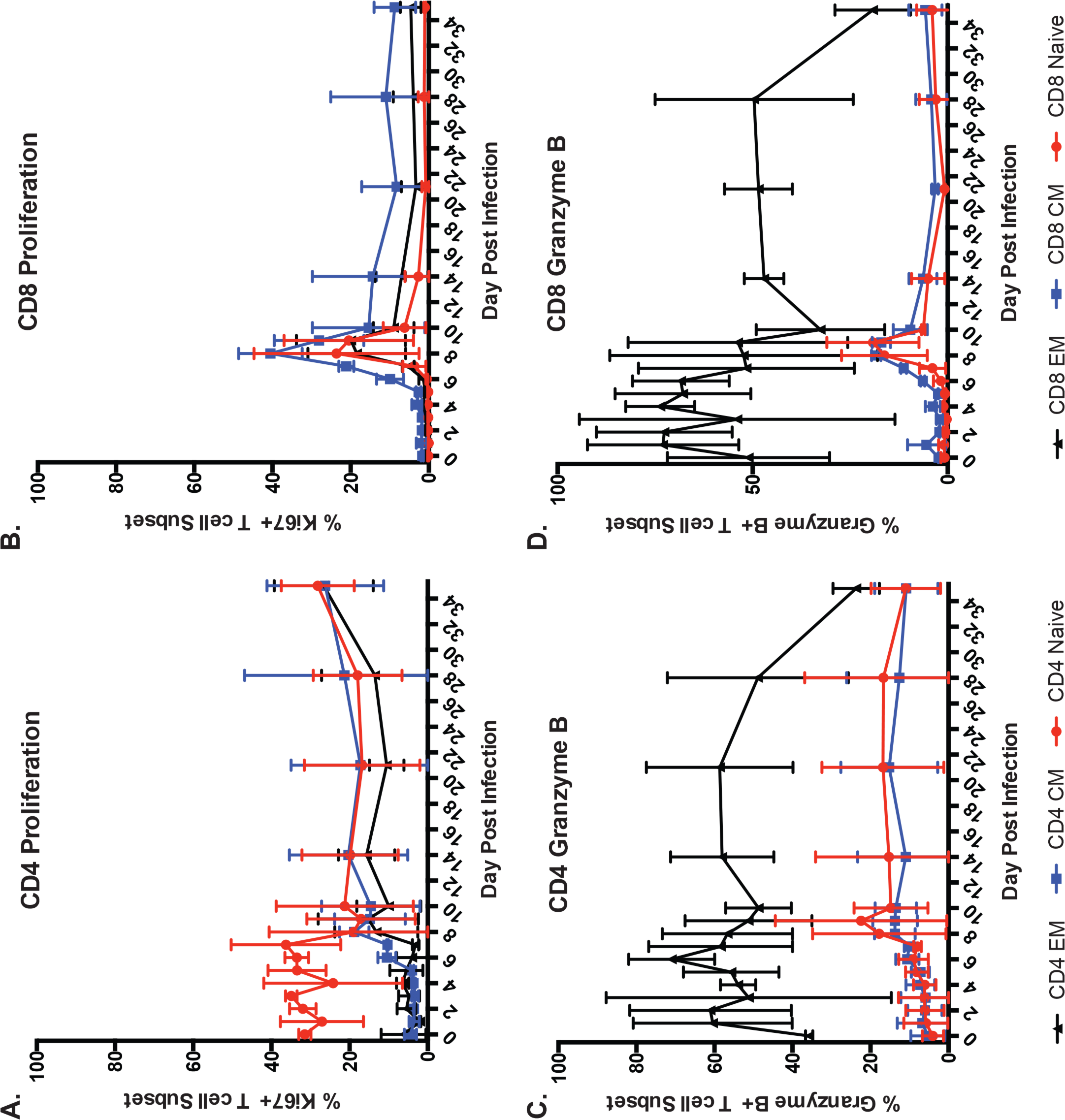

Supplement: S5 Fig — The mean frequencies of Ki67 positive (Panels A and B) and granzyme B positive (Panels C and D) T cells within Naive, central memory (CM) and effector memory (EM) subsets of CD4 (Panels A and C) and CD8 (Panels B and D) in PBMC from Animals 26021 and 26023. (TIF) [file ppat.1006219.s006.tif]

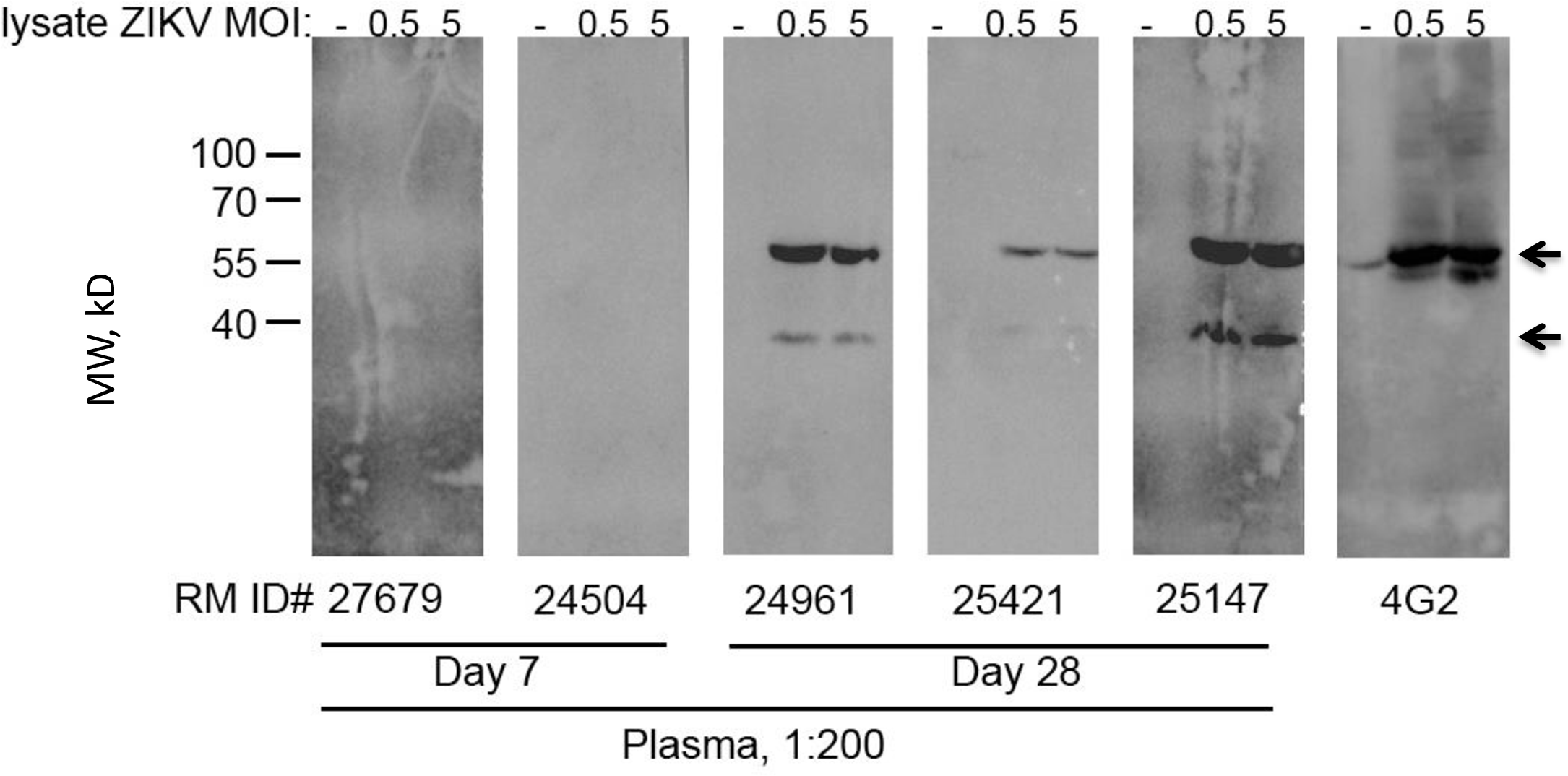

Supplement: S6 Fig — Vero cells were infected with ZIKV at MOI = 0.5 or 5 ffu/cell. At 48 h pi, cell lysates were collected, and proteins from infected cell lysates as well as uninfected Vero cells (lanes “-”) were resolved by SDS-PAGE. Proteins were probed by western blotting using plasma from indicated animals diluted 1:200, and anti-monkey Ig 2° Ab conjugated to HRP (Rockland Immunochemicals). Anti-flavivirus E mAb 4G2 was used to visualize E expression as well (right panel). Arrows indicate expected positions of E (upper arrow) and NS1 (lower arrow). (TIF) [file ppat.1006219.s007.tif]
